# Platelet-Rich Plasma in Dermatology: New Insights on the Cellular Mechanism of Skin Repair and Regeneration

**DOI:** 10.3390/life14010040

**Published:** 2023-12-25

**Authors:** Catalin G. Manole, Cristina Soare, Laura Cristina Ceafalan, Vlad M. Voiculescu

**Affiliations:** 1 Department of Cellular and Molecular Biology and Histology, “Carol Davila” University of Medicine and Pharmacy, 050474 Bucharest, Romania; 2 Ultrastructural Pathology Laboratory, “Victor Babeș” National Institute of Pathology, 050096 Bucharest, Romania; 3 Department of Oncological Dermatology, “Carol Davila” University of Medicine and Pharmacy, 050474 Bucharest, Romania; 4 Cell Biology, Neurosciences and Experimental Myology Laboratory, “Victor Babeș” National Institute of Pathology, 050096 Bucharest, Romania

**Keywords:** platelet-rich plasma, autologous transplant, skin regeneration, aesthetic dermatology, skin repairing, telocytes

## Abstract

The skin’s recognised functions may undergo physiological alterations due to ageing, manifesting as varying degrees of facial wrinkles, diminished tautness, density, and volume. Additionally, these functions can be disrupted (patho)physiologically through various physical and chemical injuries, including surgical trauma, accidents, or chronic conditions like ulcers associated with diabetes mellitus, venous insufficiency, or obesity. Advancements in therapeutic interventions that boost the skin’s innate regenerative abilities could significantly enhance patient care protocols. The application of Platelet-Rich Plasma (PRP) is widely recognized for its aesthetic and functional benefits to the skin. Yet, the endorsement of PRP’s advantages often borders on the dogmatic, with its efficacy commonly ascribed solely to the activation of fibroblasts by the factors contained within platelet granules. PRP therapy is a cornerstone of regenerative medicine which involves the autologous delivery of conditioned plasma enriched by platelets. This is achieved by centrifugation, removing erythrocytes while retaining platelets and their granules. Despite its widespread use, the precise sequences of cellular activation, the specific cellular players, and the molecular machinery that drive PRP-facilitated healing are still enigmatic. There is still a paucity of definitive and robust studies elucidating these mechanisms. In recent years, telocytes (TCs)—a unique dermal cell population—have shown promising potential for tissue regeneration in various organs, including the dermis. TCs’ participation in neo-angiogenesis, akin to that attributed to PRP, and their role in tissue remodelling and repair processes within the interstitia of several organs (including the dermis), offer intriguing insights. Their potential to contribute to, or possibly orchestrate, the skin regeneration process following PRP treatment has elicited considerable interest. Therefore, pursuing a comprehensive understanding of the cellular and molecular mechanisms at work, particularly those involving TCs, their temporal involvement in structural recovery following injury, and the interconnected biological events in skin wound healing and regeneration represents a compelling field of study.

## 1. Skin Functionality, Tissue Aggression, and Soft Tissue Lesions

The skin, the body’s largest organ, is composed of three primary layers: the epidermis, the dermis, and the subcutaneous tissue [1,2]. The epidermis—the outermost layer—acts as a protective waterproof barrier and also contributes to skin colour. The epidermis of thick skin areas (the palms and soles) consists of five sublayers: the stratum basale, stratum spinosum, stratum granulosum, stratum lucidum, and stratum corneum. The epidermis elsewhere on the body typically consists of four layers, lacking the stratum lucidum. Beneath the epidermis, the dermis consists of connective tissue and hosts a variety of structures including hair follicles, blood vessels, lymphatic vessels, and sweat glands. The dermis itself is divided into two distinct layers: the upper papillary dermis and the lower reticular dermis. The subcutaneous tissue (hypodermis, or fascia subcutanea) is the deepest layer of the skin, primarily made up of fat and connective tissue. This layer plays a crucial role in the thermal and mechanical insulation of the body, thus protecting underlying muscles and other internal structures.

However, the skin is a versatile organ, essential for maintaining overall health and well-being. It serves as a protective barrier against external threats such as microorganisms, dehydration, ultraviolet light, and physical injury, thus acting as the body’s first line of defence. Its cutaneous sensory functions, including the perception of pain, temperature, touch, and deep pressure, enable the body to respond to external stimuli. The skin also supports mobility, facilitating smooth movement and accommodating various degrees of stretching and bending. Additionally, it plays a key role in endocrine functions, notably in synthesizing vitamin D, essential for calcium absorption and normal bone metabolism. The skin’s exocrine functions involve releasing substances like water, urea, and ammonia and producing sebum, sweat, and pheromones. It also has immunologic roles, releasing bioactive substances like cytokines, and contributes to the body’s immunity against various pathogens. Finally, the skin is vital in thermoregulation, helping to either conserve or release heat as needed, and it plays a crucial role in maintaining the body’s water balance and overall homeostasis. The skin wound healing process is a remarkable interplay of cellular events and signalling molecules that participate in tissue remodelling. A profound understanding of this process could have significant implications for clinical medicine, particularly in those conditions that feature impaired healing. Depending on the body segment involved, the severity and the extent of the involved area, soft tissue healing can be a long, a slow, and sometimes incomplete (but constantly complicated) process, mainly regulated by the secretion and concentrations of the local growth factors (GFs). The development of new treatments such as PRP injections can enhance the body’s natural repair mechanisms and eventually help in speeding up wound healing.

**Figure 1:**
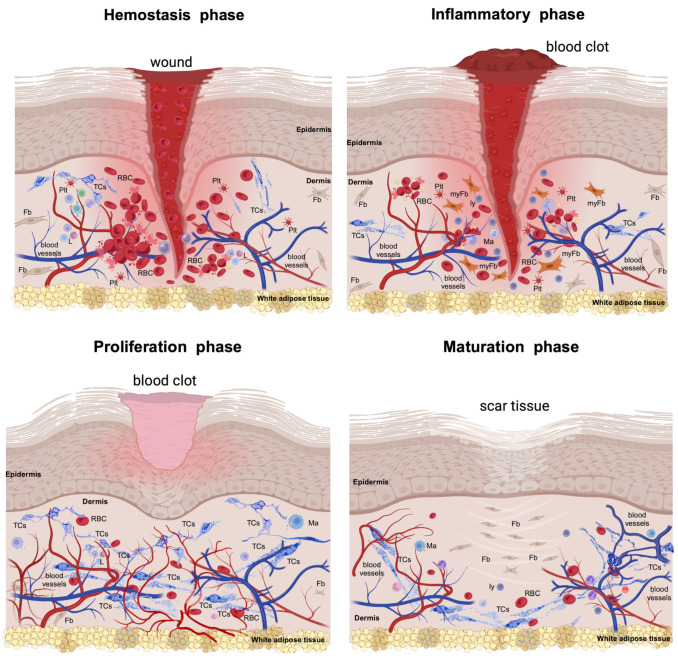
The injury of the skin triggers a series of physiologic and morphological events for repairing the injured structures. This cascade of healing falls into four overlapping phases. The hemostasis phase (primarily about coagulation) is acute and begins with injury, followed by the formation of the blood clot that dams the bloodshed; the platelets become activated by their contact with basement membranes and collagen fibres and begin aggreging. Thrombin initiates the formation of the fibrin mesh. The inflammatory phase (which is mainly about removing the cellular debris) is orchestrated by neutrophils and macrophages and their variegated secretory panel. Clinically, it lasts about 6 days and the Celsus cardinal signs feature this phase. The proliferative phase (which is about filling and covering the skin defect) structurally features the formation of the granular tissue and neoangiogenesis and wound covering by the neighbouring epithelial cells. All these intricate processes can last about 3 weeks. In both the inflammatory and proliferative phases, the formerly destroyed network of interstitial cells (including telocytes, TCs) is restored to its initial parameters. The maturation phase (dominated by structural remodelling and reorganization processes) is the longest-lasting and is dominated by the rearrangement and maturation of the collagens, increasing the tensile strength. Thus, the cellular architects of the phase are the fibroblasts (Fbs) and fibrocytes. RBC—red blood cell; Plt—platelets; L—leukocytes; myFb—myofibroblast; ly—lymphocytes. The image was created with BioRender.com.

Skin wound healing involves a series of intricate steps and sequences of multi-cellular interplays, in which secreted cytokines and chemokines ensure efficient tissue repair and regeneration (Figure 1). Usually, the wound healing process falls into four consecutive phases [3].

### 1.1. The Haemostasis Phase

This phase comes briefly after injury and is an immediate response that stops bleeding by constricting the damaged blood vessels. This process is swiftly followed by platelet activation, leading to the formation of a fibrin clot [4]. Platelets are activated, thus adhering to exposed collagen and other matrix elements, leading to the aggregation and formation of the platelet plug, stabilised by fibrin strands [5]. This clot serves a dual purpose: it halts blood loss and creates a structural framework for the next arriving inflammatory cells.

### 1.2. The Inflammatory Phase

This phase comes next and is marked by cell migration (mainly of neutrophils and macrophages). Neutrophils are the first responders, chemotactically attracted to the wound, and warding off any potential bacterial infections (mainly by phagocytosing debris and bacteria) [6]. Between 48 to 96 h post-injury, in the wound site the dispatched monocytes are differentiating into tissue-activated macrophages [7]. These cells release cytokines that further modulate the inflammatory response [8]. Additionally, the innate immune response of the adaptive immune system of the body comes into play. The key cellular players from this system (e.g., Langerhans cells, dermal dendritic cells, and T cells) are triggered into action to tackle both self- and foreign antigens present at the wound site. Recently, there has been keen scientific interest in discerning which exact cellular subset(s) is majorly dominating the cellular debris removal wound scene, or which cells are more focused on quelling infections [9]. As the inflammatory phase wanes, neo-angiogenesis takes centre stage. This sequence is marked by the proliferation and migration of endothelial cells, forming new blood vessels. Simultaneously, local pericytes provide the scaffolds and structural integrity for the burgeoning endothelial cells [10,11]. Current studies are attributing their enhanced adaptability to pericytes, considering them mesenchymal stromal cells [12]. Moreover, the formation of new blood vessels is also attributed to progenitor cells from the bone marrow [13,14,15].

### 1.3. The Proliferative Phase

This is marked by fibroblasts’ migration and the deposition of the extracellular matrix (ECM), promoting a developing cellular microenvironment. The epithelialization process permits keratinocytes to proliferate and migrate over the wound base [16]. In this stage, the dedifferentiation into the interfollicular epithelium of the Gata6+ cells originating from the sebaceous duct allows them to acquire the self-renewal capacity and further differentiate, more than they normally would, into numerous cell lineages [17]. An important aspect of the epidermal layer restoration is the regeneration of skin appendages [11,18]. It is worth noting that these epidermal stem cells predominantly display unipotency under regular conditions. This episodic plasticity enables them to differentiate into a variety of cell types, facilitating epidermal repair.

The local native fibroblast population is thriving in this phase, and they are being transferred to the newly formed clot, thus participating in the formation of the contractile granulation tissue. Certain fibroblasts undergo differentiation into myofibroblasts, playing a pivotal role in contracting the wound edges [19,20].

### 1.4. The Maturation Phase

This final phase is dominated by the remodelling, rearrangement, cross-linking, and maturation of the collagen fibres, increasing the collagen tensile strength, and strengthening the scar’s physical resistance. The wound continues to contract as the myofibroblasts remain active. Usually, unemployed cells, including fibroblasts, myofibroblasts, and inflammatory cells, undergo programmed cell death [21].

A compromised wound healing process features the evolution of chronic wounds [22]. Such wounds are predominantly seen in individuals with diabetes, vascular disorders, hemoglobinopathies, neuropathies, and in the elderly. This wound mismanaging is usually marked by recurrences, or can even require dramatic solutions (e.g., limb amputation), while severe cases may prove fatal. Metabolic diseases (e.g., obesity or type 2 diabetes), are marked by inflammatory cells within the granular tissue layer gaining immunomodulatory significance, which can further modify the wound healing course [23]. However, in different scenarios, the healing process efficiently restores the skin’s barrier function and nearly reinstates its original tensile strength. However, there is a distinction between prenatal and adult wound healing. While prenatal wound healing is mainly regenerative (mirroring the original structure of the skin), the latter often culminates in a fibrous scar, acting as a swift fix for the wound [24]. Exuberant scarring and collagen overproduction can tip the balance and frequently causing hypertrophic scars, or even keloids [25]. Emerging research suggests that scarring outcomes might be influenced by varied cellular reactions to the mechanical stress experienced by the healing skin [26,27].

## 2. Platelet-Rich Plasma (PRP): Its Introduction in Tissue Repair

Lesion progression in skin ageing and in senescent skin transformation appears due to either endogenous/(epi)genetic factors and/or exogenous factors, all of these inducing progressive accumulations of (ultra)structural skin defects secondary to their action [28]. Since photoaging impedes the normal biosynthesis of collagen and elastic fibres within a spectrum of the also-generated dermal inflammatory context, the UV radiation increases the metalloproteinases’ (MMPs) activity due to dermal extracellular matrix degradation (this including the drop-off in collagen production) [29].

The usage of Platelet-Rich Plasma (PRP) is a regenerative medicine procedure that stimulates the healing response of the tissue (after injury) using products from the blood of the patient, collected after centrifugation of a whole blood sample. Apparently, PRP is the simplest, least invasive, and most effective procedure that locally supplements the GF through an autologous transplant of GFs, thus boosting the healing processes. The PRP products have heterogeneous biological effects and a regenerative capacity, known as orthobiologics when used, for example, in treating musculoskeletal diseases [30]. The PRP targets different cells and tissue environments, modulating cell signalling and/or having a paracrine effect [31]. The main advantages of autologous PRP are, with the proper ratio of GF, the lack of immunological problems and transmitting diseases, and its ability to form a gel when injected. This low viscosity gel state is acquired within tissue, permitting platelets to locally deliver and to maintaining high concentrations of GF [28]. The anti-inflammatory effect of PRP seems to be induced by the suppression of cyclooxygenase and production of prostaglandins and by increasing the intracellular expression of inflammation-controlling cytokines [32].

PRP has been applied in human clinical settings since the 1970s because of its therapeutic properties due to the autologous GF and secretory proteins it contains and their great potential to amplify the cellular healing processes [33]. Moreover, PRP promotes the attraction, multiplication, and transformation of those cells essential for tissue repair/regeneration [34]. Products associated with PRP are sometimes referred to as a platelet-rich concentrate, platelet gel, GF-rich preparations, and platelet release, and have been previously examined into in vitro and in vivo, as add-on procedures to surgical interventions [35]. This diversity of PRP products should be considered when analysing and comparing the results of PRP transplants. Usually, these differences of various amplitudes are in accordance with the processing and leveraging of PRP products for their usage [36].

The composition of PRP can vary based on the equipment and methodology employed, leading to different levels of plasma, erythrocytes, white blood cells, and platelets in the final product. For a sample to be truly designated as “platelet-rich”, its platelet count should exceed the baseline concentration in whole blood by, as is commonly accepted, at least five-fold [37].

The PRP preparation obtained by centrifugation of the whole blood contains more than 1100 different proteins (including GFs), many of which have already documented functions in various biological processes (including regeneration/remodelling processes, or angiogenesis) [28]. PRP that boasts a platelet count of a minimum of 1,000,000 platelets/μL in a 5 mL plasma sample has been linked to improved healing [38]. PRP’s potential to accelerate healing arises from its delivery of a diverse array of GFs and cytokines, found in the α–granules within platelets. Notably, PRP exhibits an eight-fold surge in GF levels compared to whole blood [39]. Platelets degranulate within the first 10 min of participation in the clotting cascade or on contact with the basement membrane [36].

After the transplantation of PRP, the myriad of proteins and GFs either boost some normal homeostatic processes or create local premises and further scaffolds for the reparatory processes/remodelling processes [28]. However, previous observations have indicated that the efficient and short epithelialization of skin wounds is proportional to the density and synthetic activity of dermal fibroblasts [32].

In vitro studies have revealed a direct correlation between platelet concentrations and the proliferation of human mesenchymal stem cells, fibroblast growth, and the synthesis of type I collagen, thus amplifying the wound healing process [40]. Experimental animal studies have documented, mainly clinically, its efficacy in both soft and hard tissue repair [41,42].

Currently, PRP is successfully used, with remarkable results (especially in bone renewal, or soft tissue maturation) in orthopaedics, periodontics, maxillofacial surgery, urology, and plastic surgery. Yet, the medical community has different opinions regarding its efficacy, since there are studies indicating substantial upticks in bone development and maturation [35], while others found no discernible enhancements [43].

## 3. Potential Mechanisms Underlying PRP’s Effects

PRP is recognized for its youth-preserving effects in dermatology and skincare. Although the precise process by which PRP works is not fully understood, it is known that PRP injections trigger a cascade of cellular activities in aged skin. Compounds like cellulose, fibronectin, and vitronectin within PRP form complexes with growth factors at the site of injection [44]. These compounds may provide a framework to support the growth of new cells and tissues, thus aiding in the rejuvenation of aged skin through the stimulation of DNA synthesis and gene expression at the cellular level [28,45]. The application of PRP to aged skin has been shown to thicken the reticular dermis due to increased collagen and elastin fibre deposition. Additionally, laboratory studies suggest that PRP can mitigate skin damage from UV exposure by reducing MMP-1 and tyrosinase levels while boosting the production of tropoelastin and fibrillin [28].

It is recognized that, within PRP, the platelets are dormant until administered to a damaged area. Here, interaction with the tissue factor initiates a transformation in platelet form, leading to the formation of extensions known as pseudopods, which facilitate the clustering of platelets [46]. These activated platelets then secrete growth factors such as PDGF, VEGF, TGFβ, and EGF at the injury site, which contribute to a cascade of biological activities that stimulate new blood vessel formation, tissue and bone repair, cell multiplication, and overall healing [47].

PDGF is a type of glycoprotein released when platelets degranulate at an injury site. It binds to specific receptors on the membranes of target cells, leading to the creation of high-energy phosphate bonds. These bonds then activate signalling proteins that trigger the target cells’ specific functions such as cell division, the formation of new blood vessels, and the activation of macrophages [47]. TGFβ, another compound released by platelets and macrophages, serves as an inhibitor of cell multiplication in normal epithelial cells and functions both through paracrine and autocrine mechanisms [48]. Its primary target cells include fibroblasts, marrow stem cells, and pre-osteoblasts, which are integral to long-term healing, bone restoration, and skeletal remodelling. TGFβ also plays a role in preventing the formation of osteoclasts. VEGF, which was first identified as a factor that makes blood vessels more permeable, is a signalling protein that encourages the formation of new blood vessels and the development of vascular systems [49]. Lastly, EGF is a growth factor that promotes cellular growth, proliferation, and differentiation by interacting with its receptor, EGFR [50].

Platelets are small cellular fragments circulating in the blood which have a relatively short lifespan. A normal body produces around 100 billion new platelets daily [51]. In comparison, various cell types have much longer lifespans; fibroblasts can survive up to 270 days in lab conditions [52], osteoblasts range from 1 to 200 days, bone lining cells can last between 1 to 10 years, and osteocytes may endure for as long as 50 years [53]. These longer-lived bone cells may accumulate substantial cellular damage over time, contributing to senescence and the Senescence-Associated Secretory Phenotype. Conversely, the frequent renewal of platelets, occurring every 7–10 days, suggests they are less prone to the effects of senescence. The secretion of growth factors and cytokines from platelet granules is known to expedite tissue healing, a process that is augmented when interacting with the fibrinolytic system [54]. This could explain one of the pathways through which PRP exerts its regenerative and potentially anti-ageing effects.

## 4. Thrombocyte’s Genesis and Phenotype

Platelets represent fragments of the cytoplasm and cell membrane of the thrombocytogenic megakaryoblast. Platelets are the first blood figurative elements that arrive at the tissue lesion site, and are involved in the initial inflammatory phase of the healing process through their adherence, aggregation, participation in clot formation, and promotion of angiogenesis and tissue repair/regeneration by releasing proteins and growth factors [36]. Platelets produce and locally deliver antibacterial proteins, metalloproteases, coagulation factors, and membrane glycoproteins [55]. The α–granules from the platelets harbour diverse GFs, among them the platelet-derived GF (PDGF), transforming GF β1 (TGF-β 1), vascular endothelial GF (VEGF), basic fibroblast GF (βFGF), and epidermal GF (EGF). The main body of granular GFs is mainly released within the first hour, but their release continues for the next 7 days after their activation at the clot site. The dense granules of platelets contain ADP, ATP, calcium ions, histamine, serotonin, and dopamine, which are active in tissue modulation and regeneration [56].

### 4.1. Development of Platelets

The development the hematopoietic stem cells is successively located in the yolk sac, liver, and spleen. During the whole process, stem cells lose their self-renewal capacity, and they transform into short-term self-renewal cells and pluripotent progenitor cells that will initiate lymphoid and myeloid haematopoiesis. Pluripotent hematopoietic progenitors found within the proximal osteoblastic niche of the bone, due to endomitosis after multiple DNA replications (but no cytokinesis), generate the committed megakaryocytes (MKs). Their differentiation into MKs is regulated by interleukins (IL-3, Il-6, IL-11) or the stem cell factor (SCF). At the level of bone marrow, MKs are in close vicinity to bone marrow sinusoids. MKs begin the rapid development of a very dense invaginated membrane system [57] and the agglomeration of proteins either in the cytoplasm or in the granules that will become essential for further functionality of the platelets—the α– and δ–granules [58]. The hematopoietic progenitor cells are positive for CD34 and CD41. However, an increased expression of CD41, along with CD42 and CD61, indicates a commitment to the MKs’ lineage [59,60].

On the other hand, switching off the chemotactic CXCR4 signalling pathway in the development of MKs permits MKs to leave the bone marrow, and to enter mainstream circulation [61]. Thrombocytogenic MKs represent less than 0.1% of the cells in bone marrow cell populations, but this number can be altered by chemokines, thrombopoietin, and other ligands of MKs receptors [62].

However, it is known that intact MKs can cross the sinusoids walls, enter the blood stream, and create blockages at the level of the pulmonary microcirculatory sieve (another acknowledged site of platelet production) [63]. Since more than 100 years ago, when MKs were identified in the pulmonary circulation as having migrated from the bone marrow into the bloodstream and having hitchhiked into the lung circulation, the production of platelets within the lungs has been documented (especially during foetal development) [64], even though the lungs are not the primary sites of platelets’ synthesis. It was previously acknowledged that 25 × 10^4^ MKs are getting into lung tissue, hourly [60]. Already published morphometry data documented a presence of 14–65 MKs/cm^2^ in the lung (which were denser in the central and right upper lobes) [65]. However, MKs are almost 10 times more concentrated in the pulmonary artery than the aorta [66].

The development of MKs can be divided into three stages: the pro-megakaryoblast (first recognizable precursor), the megakaryoblast, and the promegakaryocyte (which, by endomitotic cell divisions, undergo DNA replication without cytokinesis, becoming the polyploid MK) [67]. Previously published studies have indicated a finite number of the endomitotic divisions of the mononuclear MKs (usually between 2–6 endomitotic cycles). Thus, many definitive MKs have a 16n of DNA content, thus indicating that the third endomitotic cycle is the most frequent [68,69,70]. The evaluation of the biological significance of polyploidisation indicated that 16n MKs will produce approximately 2000 platelets, 1 × 10^3^ more platelets than a 2n MK; this ontogenic advantage creates more efficient platelet production [71]. MKs are producing platelets within the bone marrow, but they can migrate and produce platelets in the bloodstream and could even initiate platelet biogenesis in other tissues (e.g., the lungs) [72].

After endomitosis, the MKs’ cytoplasm will definitively mature by accumulating platelet-specific proteins and developing a prominent organelle compartment and an elaborate membrane system. This invaginated membrane system was proposed as the origin of MKs pseudopodia—the proplatelets [73]. Supplementarily, a sign of MKs’ maturation (including the maturation of the MKs’ cytoplasm) is the development of an extensive three-dimensional network of flattened cisternae and tubules of different lengths and calibres, delimiting the so-called “platelet territories”. These will serve as a membrane reserve for the additional proplatelets [74]. It is easy to understand that this invaginated membrane system represents the origin of the later mature platelets, of the open canalicular system—a network of intra-platelet surface-connected membrane channels along the dense tubule system [75].

Another essential change for platelet formation is the structural, ultrastructural, and biochemical changes within MKs, including the dimerizing α– and β– 1 tubulin and their polymerisation into microtubules organised in bundles with a cortical arrangement within MKs. The bundles of microtubules are dictating the length of the proplatelets, either through sliding antiparallel microtubules (by cytoplasmic dynein), or by the localised polymerization of microtubules within the proplatelets [76]. Moreover, the subsequent proplatelet branching is also dependent upon the actin filaments’ rearrangements (as actin is a MKs cytoskeleton component). Naturally, any defects of the cytoskeletal actin dynamics are also associated with impaired platelet production in mammals (including humans) [77].

MKs proplatelets protrude through bone marrow sinusoids walls and release fragments—the pre-platelets (that are usually larger than typical platelets)—within their lumens. The pre-platelets are larger discoid cellular fragments that, via forces applied across the microtubular system, are converted into barbell-shaped platelets, or can fragment directly into platelets [60]. However, the final stages of the maturation and recovery of the platelets occur outside the bone marrow microenvironment, but exclusively within the microcirculation [78]. The proplatelets can be separated into individual platelets by centrifugation of the blood, due to applied gravitational/shear forces [79].

The progression of platelets’ formation and maturation are marked by a series of changes in the organelles’ presence and distribution within platelets, perhaps due to cytoskeletal changes. Mitochondria are (almost) twice as numerous in platelet intermediates than resting platelets, mainly representing an energetic substrate for protein synthesis in the intermediate stages of platelet development [80]. Also, dense (δ–) and α–granules are scant within the cytoplasmic bridges of barbell-shaped proplatelets [81]. Published data have documented the different intermediate stages in platelet maturation, including the resting platelets, circular pre-platelets, oval-shaped pre-platelets, barbell-shaped pre-platelets, multibody pro-platelets [79].

Since platelets are only cellular fragments of MKs’ cytoplasm enwrapped in fragments of the MKs membrane, they have no nucleus, but only scarce mRNA and a few related organelles involved in protein production (endoplasmic reticulum/dense tubular system (DTS), Golgi apparatus, mitochondria, autophagosomes, or endosomes) [82,83,84,85]. Platelets maintain the protein synthesis ability (e.g., P-selectin, actin, etc.), or can incorporate proteins from the plasma or other sources, storing all these mainly into the α–granules [86,87]. Usually, the protein content of the platelets can be modulated by protein post-translational modification and is also regulated by different rates of protein degradation (an important process for platelet function) either by the ubiquitin-proteasome system or the autophagy-lysosome system [88,89]. The total number of proteins within platelets is known as the platelet proteome (mainly produced in MKs and transferred to platelets), and this proteome contains approximately 4000–5000 distinct proteins [90,91,92].

There are few accepted mechanisms of platelet production, specifically:

1. Platelet budding from the megakaryocyte surface (apparently, these membrane blebs do not contain organelles) [93];
2. Proplatelet formation, elongation, and branching is the process by which MKs form thin cellular prolongations with a moniliform aspect, typically presenting an alternation of dilated platelet-sized segments bounded by thin cytoplasmic bridges. The rupture of these cytoplasmic bridges releases fragments of the MKs’ cytoplasm (enwrapped in MK cell membrane) into circulation, also known as platelets [94];
3. MKs cytoplasm fragmentation through the invagination membrane system (this functions as a reserve that is providing membranes for the proplatelets’ development) [95].

The in vitro production of platelets prompts reprogramming pluripotent stem cells to obtain a new expression phenotype for a few reprogramming transcription factors (Klf4, Sox2, Oct3/4, and c-Myc) [96]. It was shown that applying CD34-positive human cord blood cells to hydrogel scaffolds coated with fibronectin and thrombopoietin increases human platelet production [97].

Cultured MKs, in the presence of thrombopoietin, underwent a precise sequence of subcellular changes that finally lead to the pro-platelets’ production: *a*. the alignment/dilation of the demarcation membranes; *b*. the unfolding of cytoplasmic sheets and extensions of cell processes; *c*. the delimitation of pro-platelet-like territories; *d*. the appearance of the irregular constriction points and longitudinal axis of microtubules; *e*. the appearance and development of a central vacuole within the constriction zone centre; and *f*. platelet formation. Apparently, unlike thrombin-activated cultivated MKs, tissue MKs are surrounded by numerous shed microparticles of 100–300 nm which are strongly expressing GpIIb-IIIa, this fact supports the theory of cross-sectioned neighbouring cells processes [63,68,98,99]. MKs’ proplatelet-like cellular prolongations could be influenced by either by α–/β–tubulin polymerization inhibitors, or by low temperature [100]. Phase-contrast microscopy showed that within a single proplatelet cellular process of MK, when a more proximal segment is budding, several-times-larger platelets (about 10 mm) are obtained (in comparison with distal segment budding) [101].

Circulating platelets have a lifespan of about 8–10 days in the bloodstream. They are involved in regulating innate immunity and neo-angiogenesis. To maintain a constant count of platelets, almost 1 × 10^11^ of platelets are produced daily. They respond to blood vessel lesions by modifying their appearance, excreting their granular content, and participating in blood clot formation [62].

### 4.2. The Platelet’s Structure and Ultrastructure

**Figure 2:**
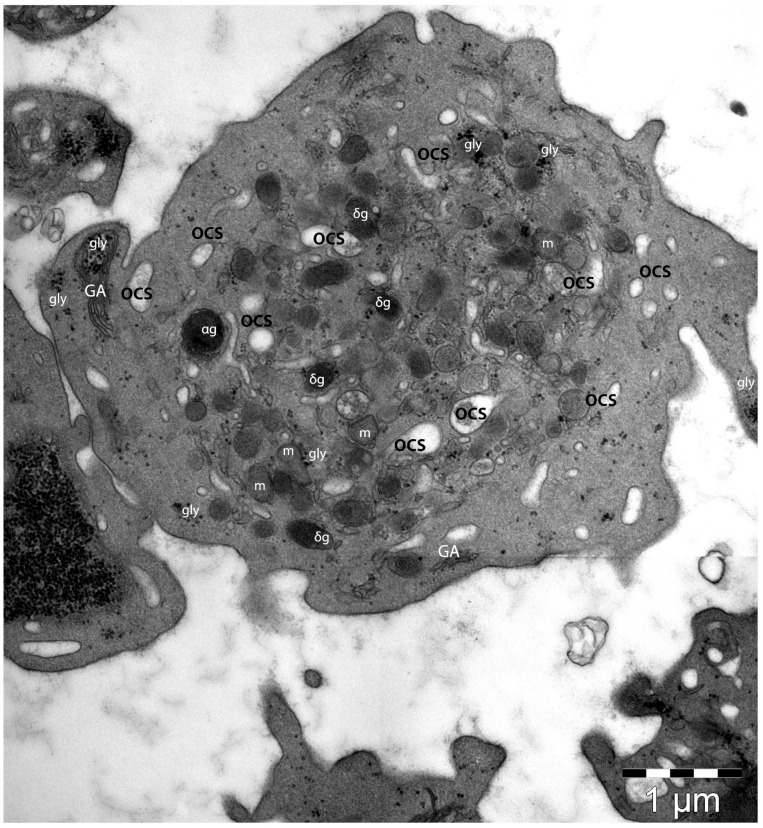
Transmission electron microscopy image of a platelet. Under the plasma membrane normal platelet specific organelles are present. The entire volume presents scarce α–granules (**αg**) and δ-granules (**δg**) among several mitochondria (**m**) and multiple granules of glycogen (gly). The elements of the open canalicular system (**OCS**) indicate its three-dimensional distribution within the entire platelet volume. A scant distribution of Golgi apparatus (**GA**) elements is also visible. (*Courtesy of Dr. E.T. Fertig, “Victor Babeş” National Institute of Pathology, Bucharest, Romania*).

Platelets are abundantly present within peripheral blood circulation and are traditionally responsible for maintaining the equilibrium between thrombosis and haemostasis. There are two functional states of normal platelets: the non-activated (resting) platelets, which maintain their discoid shape, and the activated platelets, which have morphological changes, mainly mediated by their cytoskeleton components, and which are ultrastructurally more amorphous [102,103,104]. In light microscopy, platelets demonstrate a discoid shape with dimensions 2–3 μm, but the diameter and the shape of the platelets are variegated. They usually contain α–granules, δ–granules, an open canalicular system, and microtubules, usually located on a side of the platelets, or transversely (corresponding to the constriction points of the proplatelets) [105]. The location of the microtubules (shaped like a coil) close to the constriction points could support the idea of the increased local rigidity of the membrane with implications in the clefting progression and further detaching of the platelets [106,107]. As cellular fragments, platelets are naturally marked by a plasma membrane enclosing a small aliquot of cytoplasm (Figure 2). Ultrastructurally, the plasma membrane is harbouring the apertures of an open canalicular system [108].

Biochemically, the platelet’s plasma membrane has the same composition as the MKs’ plasma membrane. Calcium release and the plasma membrane proteins participate in maintaining sphingolipids’ asymmetry with implications of their roles in thrombin generation by providing an optimum membrane surface for coagulation cascade complexes [109,110,111]. This process is involved in the coagulation cascade. The lipidomics of the platelet’s plasma membrane indicated the presence of the phospholipids (phosphatidylserine, phosphatidylethanolamine, phosphatidylcholine), sphingomyelin, and cholesterol, with this composition being crucial for the normal function of the platelets, with implications in their activation, degranulation, and exocytosis [108]. Previously published data has indicated that particular changes in lipid fractions at the level of the platelets’ plasma membrane have affected the platelets’ functioning in several studied pathologies (hypertension, lung cancer, or alcoholic liver disease) [112], or drug-related activities (aspirin, ticagrelor, or few statins intake) [113,114].

The submembranous region is one of the most important ultrastructural elements for proper platelet function, since at this level the intracellular domains of the transcellular receptor proteins interact with the proteins in platelets’ signalling cascades [115,116].

The cytoskeleton of the resting platelets is made of actin, tubulin, and spectrin; all these three conjugated proteins provide shape and support for the platelet’s morphology within circulation. This cytoskeletal architecture also permits a rapid response of the platelets to vascular damage [79]. The membrane skeleton-associated spectrin is connected to actin filaments via filamin, thus is creating a continuous ultrastructural element that promotes similar sizes for the forming platelets [117].

**Figure 3:**
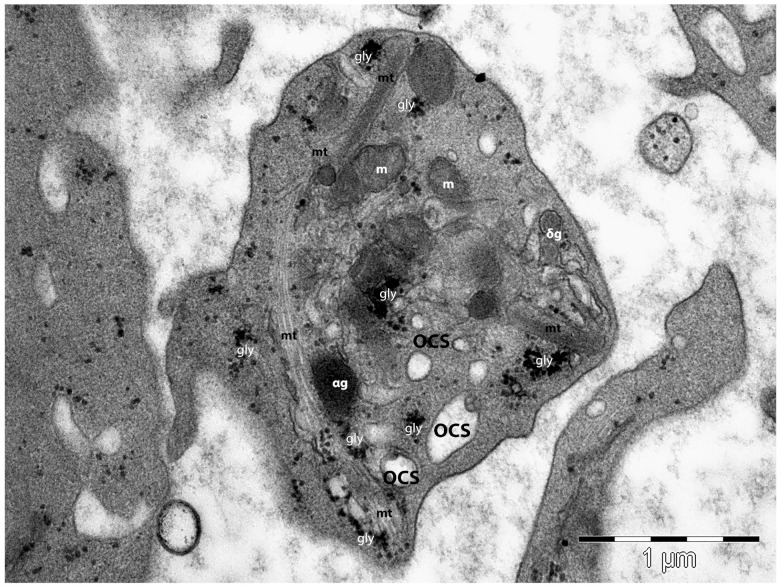
Transmission electron microscopy image of a platelet. The peripheral distribution of the microtubular (**mt**) loop is obvious and demonstrates its implication in platelet morphology and shape maintenance. Three-dimensionally open canalicular system (OCS) elements are present, along with glycogen granules (**gly**), mitochondria (**m**), α–granules (**αg**), and δ-granules (**δg**). (*Courtesy of Dr. E.T. Fertig, “Victor Babeş” National Institute of Pathology, Bucharest, Romania*).

Microtubules are polymers of α– and β–tubulin subunits and they are dynamic and polarised structures, circumferentially organised in bundles of about 20 microtubules (Figure 3). The microtubules are located below the spectrin membrane skeleton. This microtubular loop plays an important role in realising and maintaining the shape and size of the resting platelets [118]. In early platelets, the microtubules are organised longitudinally, favouring their elongated shape and increased flexibility, differently from older round platelets, whose appearance is marked by circumferentially organised bundles of microtubules [100,119].

The actin filaments are organised in a meshwork and are also dynamic and polarised fibrillar structures, whose assembly is regulated by many actin-binding proteins. The constituted platelet actin cytoskeleton provides structural support for the platelets, particularly when they are subjected to shearing forces in circulation [120,121,122]

Elements of the cytoskeleton are involved in the regulation of the platelet’s granule secretion, since, in the platelet resting state, the actin functions as a barrier to secretion but supports the granule sorting and exocytosis in the activated platelets [123,124,125,126,127].

The surface-connected canalicular system represents a three-dimensional labyrinthine system of randomly organised platelet-internal canaliculi, which originates from the plasma membrane [62,75,128,129,130,131,132,133]. The origin of this open canalicular system is related to the demarcation membrane system of MKs, and its biochemical structure is like that of the plasma membrane [134,135,136]

Below the plasma membrane, the platelets’ organelles are represented by all three types of secretory granules within (α–, δ–, and lysosomes), which are produced by the Golgi apparatus of the MKs, a dense tubular system (an inner smooth endoplasmic reticulum membrane system which initiates and modulate platelet activation), and the open canalicular system (a surface-connected extension of the plasma membrane), which helps to convey and distribute external substances into the platelets or helps to discharge α–granules [137].

However, the most prominent organelles are the α–granules. They are produced synthetically (by the trans-Golgi network), or by endocytosis (by MKs’ endocytic abilities) [138]. Ultrastructural studies indicated a heterogeneous population of α–granules within one platelet [137], even though scanning electron microscopy suggests they are homogenous ovoid alpha granules of about 200–500 nm [139]. The alpha granules have a different cargo load, which is released by platelets in a kinetically heterogeneous way [140,141,142]. Proteomic studies (including mass spectrometry) have identified several hundred (generally accepted to be over 300 proteins) soluble proteins within the α–granules, including the α–granules’ specific proteins [138,143,144,145]. Among them are some mediators of blood coagulation; the von Willebrand factor, fibrinogen, factors V, XI, and XIII, prothrombin, plasminogen activator inhibitor-1 (PAI-1), thrombospondin (TSP1), etc. [138,144,146,147]. Thus, the α–granules are involved in maintaining the blood homeostatic balance, as they are involved in regulating bleeding diathesis via the pro- and anticoagulant proteins stored within their granules [148]. Moreover, the involvement of α–granules within inflammatory processes was previously documented. The α–granules contain P-selectin and several chemokines (e.g., CXCL1, CXCL4, CXCL7) that either mediate the interaction between platelets and endothelial cells or are involved in platelets’ activation or granular content secretion, or maintain the inflammatory state.

Notably, the α–granules contain a few very important proangiogenic molecules (e.g., angiopoietin, CXCL12/SDF-1α, and matrix metalloproteinases (MMP-1, -2, and -9)), but also angiogenic inhibitors (Thrombospondin-1) that inhibit endothelial cells’ proliferation, promoting their apoptosis [149,150,151,152].

The dense granules (δ–granules) are platelet-specific organelles. They are smaller (approximately 150 nm) and numerically less abundant than the α–granules [153,154]. The δ–granules arise from the early endosomal network, concomitantly with the α–granules [155], and contain serotonin, histamine, uracil, guanine nucleotides, serotonin, calcium, potassium, and polyphosphates [156]. The δ–granules’ proteome contains 40 proteins, including regulatory proteins [157,158,159]. The molecules stored within dense granules are involved in thrombosis and blood homeostasis. The δ–granules’ deficiency usually affects bleeding, by affecting platelet aggregation and thrombus formation (thus increasing the bleeding tendency) [153,160,161]. Moreover, serotonin is marked by both vasodilator and vasoconstrictor properties, and is an important player in vascular tone regulation in humans [162]. However, δ–granules may be involved in promoting vascular permeability in inflammation [163,164,165].

The platelet’s lysosomes are heterogenous and, usually, their diameter ranges between 200 and 250 nm, similar to those in other cells. The lysosomal membrane has positivity for LAMP-1, LAMP-2, and CD63, and their content is embodied by degradative enzymes (the acid hydrolases) belonging to proteins or carbohydrate-degrading enzymes or phosphate esters cleavers [166,167,168,169].

To secrete their granules, the lipid membranes of the granules and the platelet plasma membrane must fuse (by means of the lipids and phospholipids), and these fusions are governed by the Soluble N-ethylmaleimide-Sensitive Factor Attachment Proteins Receptors (SNAREs) [170]. However, the granule secretion is supported by the elevation of intracellular Ca^2+^ and regulated by proteins like calmodulin and calcyclin [171,172].

Platelets are produced within hematopoietic bone marrow from MKs that have a characteristic immunopositivity for thrombopoietin, (also known as Mpl ligand, Mpl-l, or the MK growth and development factor, MGDF), CD34, and CD38 [67,173,174,175,176]. Flow cytometry confirmed the presence of microparticles and small CD41-positive cellular fragments in the vicinity of MKs’ platelet-producing cellular prolongations. They are presumed to be related to platelet activation and/or their physiology [177,178]. Platelets consist of glycoproteins (GpIb, GpIIb-IIIa, P-selectin) that are characteristically distributed at the level of the platelet plasma membrane or platelet organelles [179,180].

### 4.3. Roles of Platelets

Within their granules, platelets store proteins but mainly growth factors, and the degranulation of these promote local tissue homoeostasis, through platelets’ membrane adhesion and subsequently their aggregation, clot formation, and their granular content release to promote tissue repair/regeneration [36].

Due to the physical parameters of the blood, and their shape, platelets can be mainly found in proximity to the luminal surfaces of endothelial cells, where natural platelet inhibitors are present [75]. This localisation mediates the platelets’ tracking of endothelium integrity, allowing them to rapidly react to endothelial integrity issues through adherence, changing shape, aggregation, and the secretion of the growth factors they contain. In graft tissue, platelets can interact with the endothelium of blood vessels after the transplant, helping leukocytes to accumulate [181,182,183,184]. Through direct physical interaction, activated platelets can transfer chemokines to the atherosclerotic endothelium or to inflamed endothelial cells that can, after that, performantly attract leukocytes (mainly neutrophils) [185].

One physiologic role of platelets is to maintain blood homeostasis, as they are involved in the inflammatory response and adaptive immunity. The close interaction between the platelets and subsets of leukocytes (granular or agranular) has already been documented, as has the secondary secretion of immunomodulatory proteins [186]. The physical interaction between platelets and leukocytes is mainly mediated by P-selectin and PSGL-1; this interaction contributes to a performant leucocyte recruitment with the formation of circulating leukocytes–platelets conjugates that produce fibrin [187]. In the physical interaction of platelets with different types of leukocytes, the platelets’ P-selectin is responsible for the activation of different integrins: e.g., Mac-1 (αMβ2) and LFA-1 (αLβ2) on neutrophils, or β1 and β2 integrins on monocytes and lymphocytes. Moreover, P-selectin can produce a delayed response by triggering some gene expressions, and subsequently protein synthesis, producing, in the case of leucocytes, the acquiring of an inflammatory phenotype [188]. The modulatory effects of this interaction are reciprocal, mainly regulating cell cellular functions via proteases, oxygen radicals, or nitric oxide [189]. The platelet–leukocyte complex functions in the setting of the endothelial lesion, as this interaction is a prerequisite for the hyperplasia of the blood vessels intima after a vascular injury [190,191], or, for example, contributes to the progression of atherosclerosis or cardiac dysfunction [192].

**Figure 4:**
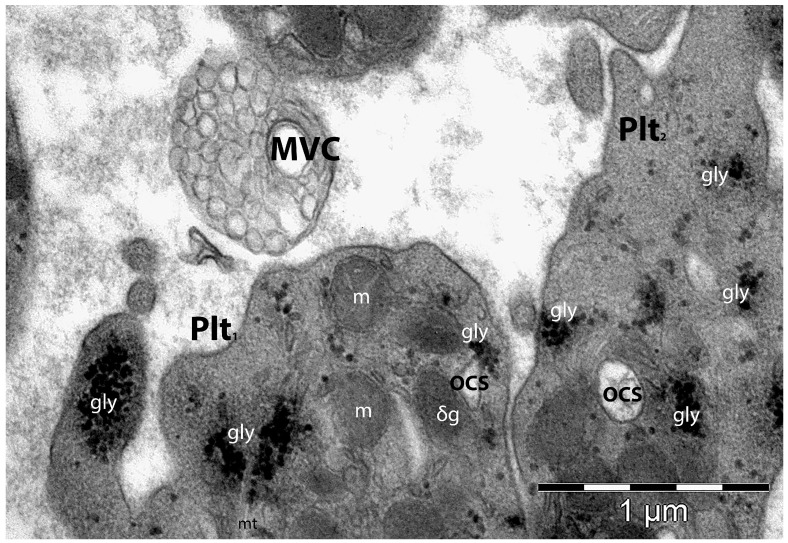
Transmission electron microscopy image of two neighbouring platelets (Plt_1_ and Plt_2_). Plt_1_ is in close proximity to a just-shedded multivesicular cargo (**MVC**)—a complex group of platelet-derived membrane-bound structures that activate and regulate intercellular pathways. MVC are vectors of transport for nucleic acids, proteins, and lipids, regulating the local microenvironment (but also remotely) with implications in cellular functions’ modulation or tissue regeneration/repair. **gly**—glycogen; **m**—mitochondria; **OCS**—open canalicular system. (*Courtesy of Dr. E.T. Fertig, “Victor Babeş” National Institute of Pathology, Bucharest, Romania*).

The secretion of the granular cargo (especially from the α–granules) contains mainly mitogenic, coagulation, and angiogenic factors, as well as inflammatory mediators. The content of the platelet’s vesicular cargo may act either locally, influencing the activity of neighbouring cells, or systemically [193]. The platelet’s extracellular vesicles are small (100–250 nm) membranous structures produced and shedded by platelets under different stimuli (Figure 4). These extracellular vesicles (cargos) contain RNA, MMPs, antigens, and cytokines [194].

However, platelets are essential for angiogenesis and maintaining blood vessel integrity via several tyrosine-based activation motifs (ITAM) that regulate cellular interactions [195]. The lipids from the structure of the platelet’s membrane (e.g., phosphatidic acid, lysophosphatidate, S1P) are cellular chemoattractants that are involved in angiogenesis [196].

Platelets can regulate blood vessel permeability. Thus, a severe depletion of platelet numbers affects the endothelium integrity, with the appearance of disruptions and fenestrations that permit red blood cells to cross into the surrounding tissue and to form petechiae [164]. The angiogenic role of the platelets is supported not only by their number, but also by their functionality, and their direct contact with endothelial cells, a process which is favoured by a series of adhesion molecules [197]. However, within platelets’ granules, among their angiogenic factors there are also anti-angiogenic factors (e.g., endostatin, thrombospondin-1, angiopoietin-1, angiostatin, etc.), contributing to the vascular homeostasis in the repairing tissue [198]. Upon platelet activation or due to oxidative stress, by membrane budding, platelets release microparticles that are mostly found at the site of angiogenesis [108,199]. Thus, they stimulate new blood vessel formation or increase vascular permeability. Platelet microparticles are also found frequently within solid tumour microenvironments, where they are involved in protein transfer or RNA/DNA transfer to endothelial cells [151].

At the level of vascular injury, platelets, via their secreted signalling molecules, are producing chemoattraction and the densification and numeric proliferation of the neighbouring smooth muscle cells, and with SDF-1α, differentiation into endothelial cells and vascular sprouting [200,201,202]. Previous data reported that there is an optimal concentration of platelets (1.5 × 10^6^ platelets/μL) that induces angiogenesis in human endothelial cells; exceeding this concentration of thrombocytes could exert an inhibition of angiogenesis [203].

Platelets are also important in lymphangiogenesis since, similar to lymphatic endothelial cells (but different from blood vessels’ endothelial cells), they have a positive membrane expression for C-type lectin-like receptor (CLEC)-2, which ligates podoplanin [204,205]. Through this, they can bind podoplanin (a type I transmembrane sialomucin-like glycoprotein), which can lead to platelet activation, but the deficiency of podoplanin impairs lymphatic vessel formation [206,207]. Platelets are also involved in the embryonic differential development of lymph vessels and blood vessels, while also maintaining this separation (e.g., by platelets’ plugs), or being involved in further lymphatic vessel-repairing processes [208,209,210].

In the process of endothelial cell differentiation, platelets act as recruiters for progenitor cells, which, after that, in an SDF-1/CXCR4 manner, become functional endothelial cells [211]. One layer of platelets could realise leukocyte recruitment or contribute (by granular P-selectin) to monocytes’ recruitment, thus it could be rational to highlight the task of platelets in supporting neointimal formation, or intimal hyperplasia [212,213,214].

Platelets also secrete numerous chemokines involved in tumour cells’ invasion, tumoral cell proliferation, or metastasis regulation [215]. Among the series of GFs that the granules contain, PDGF and TGFβ1 appear to be integral modulators [36]. Activated platelets, via the key molecules and GFs they are secreting, are contributing, along with other immune cells, to all four stages of tissue repair, specifically to haemostasis, the inflammatory/immune response, tissular proliferation, and tissue remodelling [216,217,218,219,220]. Within a lower pH micro-environment (that features the initial stages of wound healing) platelets seem to increase the concentration of PDGF and therefore stimulate the proliferation of Fb, since in the same pH range TGFbeta1 increases the production of collagen. Later, by releasing IL1, platelets mitigate inflammation, promoting dense scar formation [36].

## 5. A Concise Evolution of the PRP Therapy Concept

With comparative results to those hypothesized for stem cell therapies, PRP is quickly becoming a pillar of regenerative medicine. Its applications are expanding into diverse medical fields, such as aesthetic dermatology, orthopaedics, sports medicine, and surgical procedures.

Everything started more than a century ago, in 1842, with Donne’s ground-breaking revelation about blood containing structures beyond the erythrocytes and leukocytes [221]. It was Julius Bizzozero who initially coined the term “*le piastrine del sangue*” for platelets. In 1882, he documented the pivotal role of platelets in in vitro blood coagulation and their contribution to in vivo thrombosis. He also observed that the vascular wall could inhibit platelet adhesion [222,223]. Advancements in regenerative therapy were further made by Wright’s identification of MKs as platelets’ progenitors [224].

During the 1940s, medical professionals employed “embryonic extracts” (rich in growth factors and cytokines), to foster wound healing [225]. Successful surgical procedures rely, *inter alia*, on prompt and efficient wound healing, Thus, in April 1944 Eugen Cronkite et al. proposed the idea of using thrombin and fibrin in skin grafting [226,227]. By using these components, the firmness and stability achieved are integral to grafting surgeries.

At that time, the concept of platelets secreting growth factors in PRP and thus contributing to PRP’s efficacy emerged as a hypothesis. This theory was validated in the 1980s when it was established that the bioactive molecules (or GFs) discharged from platelets repair damaged tissues, such as skin ulcers [228,229]. Numerous studies have been published onto this topic since then. A prominent area of research in this domain has been the synergistic use of PRP with hyaluronic acid [230]. In 1962, Cohen identified the epidermal GF (EGF). This discovery paved the way for the subsequent finding of growth factors: platelet-derived GF (PDGF) in 1974 and vascular endothelial GF (VEGF) in 1989 [224]. Medical advancements, in a broader sense, have also spurred rapid progress in platelet applications. In 1972, Matras pioneered the use of platelets as sealants to maintain blood homeostasis during surgeries [231]. Later, in 1975, Oon and Hobbs were the pioneers in applying PRP in reconstructive treatments [232].

In 1986, Knighton and his colleagues pioneered the description of platelet concentrate protocols, coining the term “autologous platelet-derived wound healing factors” [233]. Following the establishment of these protocols, the technique started to become very popular in the field of aesthetic medicine [234]. Since the latter part of the 1980s, PRP has been a staple in the field of regenerative medicine and regenerative dermatology [229].

Beyond its applications in general and cardiac surgery, PRP found acclaim in maxillofacial surgery during the early 1990s. It was utilized to enhance graft integration during mandibular reconstructions [235]. The dental industry also recognized PRP’s potential. By the late 1990s, PRP began to be employed to support the integration of dental implants and support bone regeneration [236]. A related product, fibrin glue, was also introduced during this period [237]. A significant advancement in dental PRP applications came with Choukroun’s invention of platelet-rich fibrin (PRF), a unique platelet concentrate that eliminated the need for anticoagulants [233]. The 2000s witnessed a surge in PRF’s popularity, with its use spanning various dental treatments [238].

From 2010, and possibly even before, PRP has made notable inroads into cosmetic dermatology. Post-PRP injection, there is a marked rejuvenation of the skin, characterized by enhanced hydration, elasticity, and improved complexion [239]. An added application of PRP has been its use in augmenting hair growth [240]. The studies of Gentile et al. highlighted that hair density and count can be positively influenced by PRP injections [241]. Additionally, a pre-treatment with PRP prior to undergoing hair transplantation has been shown to boost hair growth and enhance the overall density of the hair [242,243].

Recent advancements in cosmetic dermatology show that integrating PRP with CO_2_ laser treatments leads to a pronounced decrease in acne scarring [244,245]. Moreover, the combination of PRP with micro-needling has been observed to produce more structured collagen formations in the skin, compared to using PRP by itself.

The application of PRP in medicine could be extensive, with notable contributions to various medical disciplines, including gynaecology [246], urology [247], and ophthalmology [248]. Overall, before mentioning the applications (and presumable benefits) of PRP in skin conditions, it is worth mentioning that neo-angiogenesis plays a primary role in the formation of the granular tissue and in maintaining keratinocytes’ survival rate. Thus, many prophylactic or therapeutic platelet transfusions are performed with activated platelets. When discussing PRP treatments, we need to differentiate between different platelet concentrates, based on their cell content and fibrin architecture, such as those presented in Table 1.

A new coding system for PRP was designed by Kon E. et al. to simplify the description of PRP characteristics and ensure consistency and clarity in its preparation and use. Here is a breakdown of the PRP coding system [251]:

1. N1N2—platelet composition:
  - The pair of digits relates to the concentration of platelets in the PRP compared to the basal levels in the blood.
  - “1” represents a concentration range of 100,000–200,000 platelets/μL.
  - “2” represents a concentration range of 200,000–300,000 platelets/μL.
  - So “12” would mean that the PRP has twice the basal concentration of platelets, and the PRP’s platelet concentration is between 200,000–400,000 platelets/μL.
2. N3N4—purity:
  - N3 indicates the absence (0) or presence (1) of erythrocytes (RBCs).
  - N4 represents the concentration of leukocytes, with “0” indicating no leukocytes and increasing numbers indicating higher concentrations.
  - For example, “11” would indicate the presence of erythrocytes and a low concentration of leukocytes in the PRP.
  - Both leukocytes and erythrocytes can affect the results obtained after PRP injections due to side effects and interferences [252].
3. N5N6—activation:
  - N5 specifies whether the PRP is endogenously activated (0) or whether it is activated externally, before PRP injection (1).
  - N6 indicates whether calcium was added for activation, with “0” meaning no calcium added and “1” meaning calcium was added.
  - For instance, “00” would mean that the PRP is endogenously activated, and no calcium has been added.
  - Previous research has indicated that the level of calcium can impact the cellular and tissue reactions elicited by PRP [253].

## 6. Utility of PRP in Dermatological Practice

### 6.1. PRP in Skin Burns

Skin burns are lesions caused by the action of high temperature agents over the skin, and can involve the epidermis only (superficial burns), the epidermis and superficial, papillary dermis (partial thickness burns), or deep skin burns (that involve the reticular dermis) [32]. The burn lesion has definitive stages of evolution: at 7 days of evolution it is characterised by acidophilic material covering the wound surface and dermal inflammation, with scarce CD34-positive cells; at 14 days, focal re-epithelization of the dermis and wound healing appear; and at 28 days a flawed epithelium with keratinocytes’ nuclear pyknosis cytoplasmic vacuolization is formed [254].

PRP plays a significant role in enhancing the healing and appearance of post-burn scars by improving pigmentation, skin texture, and alleviating associated neuropathic pain. This positive effect is primarily attributed to the growth factors in PRP, which promote neovascularization and neo-collagenogenesis, thereby aiding the healing process in burn wounds [255,256]. The application of PRP can be achieved using various injection techniques (the “linear retrograde and fanning” method, the “tunnelling” technique, etc.). Published data indicate that monthly PRP sessions over three months of treatment produced remarkable enhancements in terms of itch relief, scar pigmentation, and suppleness. The improvement in pain is believed to result from nerve growth factors present in the PRP, promoting nerve recovery and regeneration [257].

The PRP product injected in the skin burn wound, at 7 days after injection, determined the covering of the wound area by migrated spindle-shaped cells from the wound periphery with heterogeneous disorganised dermal collagen fibres [258,259]. Fourteen days after the PRP injection, the epidermis indicated a normal appearance but the dermis was the site of a prolific synthetic fibroblast-like infiltrate with an increased density of blood vessels [260]. However, the lesion injected with PRP had an increased number of CD34-positive cells at 14 and 28 days after the injection [261].

Overall, the beneficial effects of PRP administration are epithelization, endothelial cell migration, and neovascularization, increasing the production of collagen fibres and dermal matrix proteins, and improving dermal regeneration [262]. PRP upregulates the dermal expression of collagen I and downregulates the matrix metalloproteinase proteins 1 and 9 (MMP-1 and MMP-9) [263].

### 6.2. PRP in Alopecia

Hair on the scalp significantly contributes to an individual’s self-perception. Individuals with alopecia experience noticeable physical changes, leading to potential psychological distress and reduced self-confidence [264]. Hair loss, also called alopecia, stands as a prevalent concern among those seeking dermatological advice, and its duration can range from temporary to permanent [265]. Alopecia can be broadly categorised into non-cicatricial and cicatricial types. Androgenetic alopecia, telogen effluvium, alopecia areata, trichotillomania, and anagen effluvium are examples of non-cicatricial alopecies. On the other hand, conditions like lichen planopilaris, frontal fibrosing alopecia, folliculitis decalvans, and cutaneous discoid lupus erythematosus contribute to cicatricial alopecia [266].

For an effective alopecia treatment, it is crucial to conduct a thorough assessment, including an in-depth review of dietary habits, ensuring tailored patient care [267]. Clinicians have a variety of traditional treatment methods at their disposal to address alopecia. For instance, androgenetic alopecia treatments comprise topical minoxidil, oral medications like finasteride and dutasteride, peptides, and hair transplant procedures [268]. To address telogen effluvium, approaches such as high-protein diets, iron supplements, avoiding catagen-inducing medications like beta-blockers and retinoids, and treating underlying conditions that promote catagen (like thyroid issues or hyperandrogenism) are recommended [269].

With increasing interest in regenerative therapies, PRP has emerged as a promising treatment option for various types of alopecia, attributed to its high concentration of growth factors and cytokines. As it is derived from the patient’s own blood (autologous), PRP eliminates the potential adverse reactions often associated with other therapeutic methods, thus enhancing patient compliance. Moreover, because PRP operates differently from other treatments, it could further enhance results when used alongside treatments like minoxidil and finasteride [270]. PRP is derived from autologous blood and is concentrated with platelets that release various GFs, PDGF, TGF-β, VEGF, and EGF, which play a pivotal role in the hair cycle. They enhance the proliferation of dermal papilla cells, stimulate angiogenesis, and prolong the anagen (growth) phase of the hair cycle [242].

#### 6.2.1. Androgenetic Alopecia (AGA)

Commonly referred to as male and female pattern baldness, androgenic alopecia results from genetic predisposition and hormonal influences. Studies have shown that a PRP treatment increases hair density and promotes thicker hair shafts in androgenic alopecia patients. The growth factors in PRP likely counteract the effects of dihydrotestosterone (DHT), the primary hormone responsible for androgenic alopecia [271].

Singhal et al. reported, after three months of PRP treatment, that all ten patients with androgenetic alopecia displayed significant hair growth, witnessing an average 65% decrease in hair loss. Remarkably, six patients saw new hair growth in just 7 days, while the rest observed it within 15 days [272]. In another study involving 11 AGA patients undergoing PRP treatment, the hair pull test was negative for 9 patients after four PRP sessions held bi-weekly. Also, a noticeable improvement in hair thickness and fullness was reported [273]. Furthermore, Greco et al. conducted a study with 10 AGA patients—5 treated with PRP and 5 untreated. Their findings suggest that using PRP as mesotherapy in AGA results in a significant boost in both hair thickness and density [274,275].

Uebel et al. noted a marked increase in hair density and growth when follicular units were pre-treated with platelet plasma growth factors prior to implantation. A noticeable difference in the success rate of follicular units was seen when comparing treated and controlled scalp areas [243]. A post-slitting intra-operative PRP treatment was found by Garg et al. to boost hair density swiftly, decrease transplanted hair’s catagen loss, speed up skin recovery, and activate dormant follicles in follicular unit extraction transplant subjects compared to a placebo [276].

Generally, hair growth necessitates performant vascular support and the development of this could be influenced by angiogenesis due to the production of VEGF by keratinocytes and papillary fibroblasts [277]. PRP induces follicular stem cells’ differentiation, and the anagen phase’s prolongation, stimulating hair growth via upregulations of the FGF-7 and β-catenin signalling pathways [278] Previous studies reported positive therapeutic effects on hair loss (male/female pattern) after interfollicular injections of CD34-positive cells and PRP [279]. Autologous PRP seems to increase the proliferation of dermal papillary cells, while also increasing the expression of fibroblast growth factor 7 (FGF-7), β-catenin, extracellular signal-related kinase (ERK), and Akt signalling, further suggesting a mechanism for hair restoration [280,281]. Likewise, a systematic review by Leo et al. supported the beneficial effects of PRP in hair regrowth and highlighted its safety profile [282].

#### 6.2.2. Alopecia Areata (AA)

AA is an autoimmune disorder causing patchy hair loss. Trink et al. demonstrated that PRP induced significant hair regrowth in androgenic alopecia patients, potentially due to the immunomodulatory effects of the GFs present in PRP [283]. Also, evidence suggests that PRP might offer therapeutic benefits for AA patients by reducing local inflammation and stimulating hair regrowth [284].

PRP was successfully used in AA [285,286]. Administering PRP to hair skin improves the skin ischaemic conditions by increasing the density of vascular structures within the hairy skin, thus promoting cellular proliferation and differentiation [287]. Greco et al. employed PRP as mesotherapy for a single patient with AA, yielding positive outcomes after 10 months [274]. However, a comprehensive study with 45 patients demonstrated PRP’s efficacy in treating AA, showcasing significant hair regrowth, reduced hair abnormalities, and minimised scalp discomfort [283]. Singh et al.’s research revealed that of 20 AA patients treated with PRP, only one experienced a relapse, and the treatment was well-received [288]. Donovan pointed out the potential of PRP in treating specific steroid-resistant forms of AA [289]. El Taieb et al. found PRP to be more effective than topical minoxidil 5% in treating AA based on their study involving 90 patients [290].

#### 6.2.3. Telogen Effluvium (TE)

TE is often caused by stress, medications, or hormonal changes, and is characterised by diffuse excessive shedding. While fewer studies have focused on PRP’s efficacy in TE, preliminary reports suggest improvements in hair density and a reduction in hair shedding post-PRP treatment [291,292].

The efficacy of PRP in treating TE lacks concrete research evidence. It was previously documented that minoxidil increases the ratio of Bcl-2/Bax by activating ERK and Akt [293,294]. On the other hand, finasteride induces the activation and prolongation of the anagen phase of hair growth, which is responsible for the longitudinal and diametral growth of hairs, but it also induces the expression of apoptosis inhibitors and caspase [295].

Studies comparing PRP with other treatments, like minoxidil or finasteride in AA, suggest that while both treatments may offer benefits independently, the combination of PRP with traditional therapies often produces superior results [296]. Fakahany et al. observed that automated micro-needling combined with PRP effectively treats various hair loss conditions, including cicatricial alopecia [297]. The success of grafted follicles in cicatricial alopecia areas, which typically have a reduced blood flow, relies primarily on the vascular bed’s blood supply. PRP, laden with growth factors like IGF-1, βFGF, and VEGF, can ameliorate cutaneous ischaemic conditions, enhancing the hair follicles’ surrounding vascular structures [298]. Saxena et al. found that introducing 1 mL of PRP intradermally to a recipient area test patch just before grafting was effective in managing cicatricial lichen planus [299]. In cicatricial alopecia cases, the scarcity of ostia might suggest a limited potential for restoring destroyed hair follicles. Thus, new follicles from follicular unit extraction punch grafting might serve as a stem cell source, APM, and nerve bundle.

### 6.3. PRP in Skin Ageing

The relentless search for non-invasive rejuvenating therapies has brought PRP to the forefront of aesthetic dermatology and rejuvenating medicine. This autologous product from whole blood harnesses a concentrated cocktail of growth factors and cytokines, presenting a biocompatible and effective approach to combat skin ageing. The regenerative potential of PRP is mainly attributed to its rich milieu of growth factors, among them the PDGF that stimulates fibroblast growth and promotes collagen production, improving the skin’s texture and elasticity [300]. TGF-β enhances the synthesis of the extracellular matrix and plays a role in tissue remodelling [301]. VEGF boosts angiogenesis, ensuring the better oxygenation and nourishment of skin tissues [302]. EGF facilitates cell growth and differentiation, leading to epidermal and dermal regeneration [303]. FGF promotes fibroblast function and augments skin repair mechanisms [304].

All the aforementioned growth factors are only a few of those provided by the platelets within PRP. Together, they stimulate collagen synthesis, enhance tissue regeneration, reduce inflammation, and amplify skin hydration—the well-acknowledged hallmarks of youthful skin. PRP therapy has demonstrated efficacy in mitigating wrinkles and enhancing skin elasticity, possibly due to increased collagen production post-therapy [305]. PRP treatments can improve skin tone and firmness, making it a viable option for those with sagging skin [306]. With its capacity to stimulate neo-collagenesis, PRP can lead to smoother skin and a reduction in pore size [307]. PRP’s angiogenic properties can rejuvenate the skin’s microcirculation, leading to brighter, more radiant skin [308]. PRP emerges as a promising treatment modality for skin ageing, riding on its autologous nature and multiplicity of regenerative factors. However, the procedure’s success is influenced by factors like PRP preparation techniques, individual patient differences, and treatment protocols. While preliminary results are encouraging, larger-scale, randomised studies are essential to firmly establish PRP’s role in anti-ageing treatments.

Notable benefits of PRP’s administration on facial skin, like skin suppleness, texture, hydration, wrinkle reduction, and micropigmentation, were observed between 1 and 3 months post-treatment [305,309]. In one specific study, the outcomes persisted for 6 months after completing three PRP sessions, although there was a slow reversion to the original state [310]. Previously published data have indicated the successful administration of PRP as a single treatment for periorbital wrinkles and dark circles, an improvement of the perioral area, skin fitness enhancement, and a reduction in skin redness, revealed an improvement in skin texture and increased patient satisfaction for crow’s feet, and a notable enhancement in skin colour for infraorbital dark circles [305,306,309,311,312,313,314,315].

### 6.4. PRP in Acne Scarring

Acne scarring, a significant cosmetic concern, can severely impact an individual’s self-esteem. With the rise of regenerative medicine, PRP has shown promise as a treatment modality for acne scars. PRP, an autologous preparation derived from whole blood, offers a rich mix of growth factors that can foster tissue repair and regeneration. The PRP growth factors initiate collagen production, stimulate angiogenesis, and boost tissue repair, making PRP a potent tool against acne scars.

PRP has demonstrated the potential to enhance the texture and appearance of atrophic scars by amplifying collagen synthesis and remodelling [316]. PRP can be combined with other treatments like micro-needling and laser therapy to synergize their effects and achieve improved outcomes. Micro-needling with PRP has shown superior results in reducing scar depth and improving skin texture compared to micro-needling alone [317].

PRP’s anti-inflammatory properties can accelerate healing and reduce the downtime post resurfacing procedures like lasers or dermabrasion [318], thus emerging as an effective therapeutic option for acne scars, as can be seen in Table 2. Its autologous nature minimises the risk of allergic reactions or incompatibility. While initial results are encouraging, the optimal preparation techniques, concentrations, and treatment protocols are still subjects of ongoing research. Large-scale, randomised trials are warranted to conclusively establish PRP’s role in acne scar management.

### 6.5. PRP in Melasma

Melasma is a persistent hyperpigmentation disorder characterised by an overproduction of melanin by melanocytes, resulting in yellow-brown patches, particularly on sun-exposed skin (centrofacial, malar, or mandibular) [328,329]. The exact cause remains unclear, but it is believed to be a combination of factors including UV exposure, hormonal changes, inflammation, and oxidative stress that influences skin cells, leading to melanogenesis [330]. The prevalence of melasma varies largely [331]. It predominantly affects women, with a 9:1 female-to-male ratio, and particularly those in their reproductive years, pregnant, or with skin phototypes III-V [329,332]. Common treatments encompass topicals like hydroquinone and azelaic acid, systemic treatments like glutathione, and procedures like bleaching and lasers, although these treatments can sometimes lead to inconsistent outcomes and skin reactions [328].

PRP’s potential in treating melasma stems from its wound healing properties and the angiogenic effects of its growth factors, PDGF, TGF-β1, and TGF-β2. Specifically, TGF-β1’s ability to inhibit tyrosinase—an enzyme crucial for melanin production—might be key. This inhibition might be due to its effects on transcription factors such as microphthalmia associated transcription factor (MITF) and through the prolonged activation of the extracellular signal-related kinase.

However, the current literature on PRP for melasma is limited, with only a few studies including a meta-analysis, a non-randomized clinical trial, and a prospective study available for review. Even though all these studies utilised the Melasma Area and Severity Index (MASI) to assess outcomes, the treatments varied—ranging from PRP injections and tranexamic acid to lasers and micro-needling. Overall, incorporating PRP in combination treatments like fat grafting and CO_2_ lasers has shown marked progress in post-burn scar appearance [333].

### 6.6. Limitations of PRP

PRP therapy shows encouraging results in treating various skin conditions. However, there is a pressing need for extensive, randomized, controlled trials. Most existing studies are limited by small sample sizes, inconsistent criteria for preparation protocols and outcome comparisons, and brief follow-up durations. Despite numerous studies, the impact of different parameters on PRP’s clinical effectiveness in aesthetic medicine remains unclear. A primary issue in evaluating PRP’s effectiveness across studies is the lack of standardization. Another significant challenge is the inconsistency and inadequacy of assessing outcomes. Further research is essential to establish the most effective methods and procedures for collecting, processing, and administering PRP.

The significant variability in PRP preparation protocols has been a recurrent topic of discussion [38,334,335]. One reason for this variation is the array of classification systems proposed. In 2009, Ehrenfest et al. [336] introduced the first such system, categorizing platelet-rich preparations based on two factors: the presence of leukocytes and the density of the fibrin network. Thus, we can divide PRP in four categories [296]: pure PRP/P-PRP (leukocyte-poor, low-density fibrin network), leukocyte-rich PRP/L-PRP (leukocyte-rich, low-density fibrin network), pure PRF/P-PRF (leukocyte-poor, high-density fibrin network), and leukocyte-rich PRF/L-PRF (leukocyte-rich, high-density fibrin network).

Additionally, some experts advocate for using the DEPA classification system by Magalon et al. [337], which categorizes different PRP products based on the dosage of injected platelets, the efficiency of platelet recovery from blood, the purity of the PRP (ratio of platelets to red and white blood cells), and the activation process [296].

## 7. Further Perspectives

It is already (clinically) acknowledged, in few different conditions (including dermatological disorders), that the administration of PRP has tremendous clinical or physiognomic benefits. However, we presume that the roles, the mechanisms of action, and the structural advantages of PRP delivery are still waiting to be approved and intimately described. It is our assessment that PRP’s involvement in regenerative dermatology is still at the beginning of the slope of enlightenment. The simplest acceptance that PRP is just a collagen stimulator is marked by prosaicism. There is still a need for more solid data to decipher the cellular and molecular machinery that explains PRP’s clinical benefits and reveals the complexity of the intricate reparatory/regeneration mechanisms that PRP is producing.

Since the first documentation of PRP’s clinical benefits on skin repairing/regeneration to the present day, much important data have been reported as well as a few essential changes in skin structural science (with respect to cellular and molecular dermatology). Presumably, some of the changes are still hiding those rational explanations for the PRP advantages that should be considered. Indubitably, the local application of trophic factors within the dermis promotes cell division and the synthesizing of cellular components of the dermal microenvironment, structurally and secondarily impacting the epidermis.

**Figure 5:**
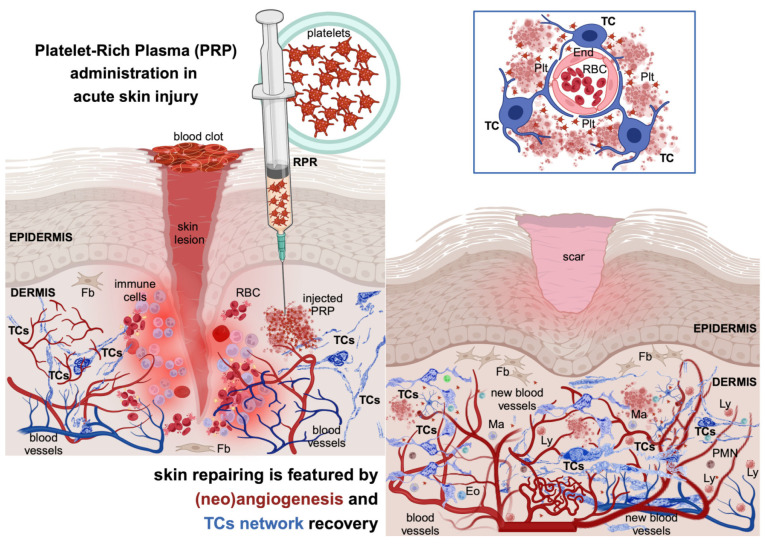
Providing a platelet-rich plasma (PRP) treatment to the site of the skin injury helps the structural recovery in terms of speediness and the performant regeneration of affected cutaneous components. Considering the myriad of growth factors and cytokines, it is attractive to presume that PRP is eliciting the vascular recovery of telocytes (TCs), cells with a documented angiogenic potential within the interstitia of other organs. Inset: platelets could stimulate the involvement of TCs (by their cellular prolongations—telopodes) as nursing cells for the new-formed blood vessels. Plt—platelets; RBC—red blood cells; End—endothelial cell; Ma—macrophage; Fb—fibroblast; Ly—lymphocyte; Eo—eosinophil; PMN—neutrophil. The image was created with BioRender.com.

At least for skin science (but many times proved within the stroma of other organs), the presence of a new population of interstitial cells has already been identified—telocytes (TCs) [338,339,340]. A great body of already published data has (ultrastructurally and immunohistochemically) described skin TCs and also documented the involvement of TCs in neo-angiogenesis and in the tissular reparatory/recovery/remodelling/regeneration process [340,341,342]. However, to date, those solid structural and ultrastructural studies of tissue regeneration/repair after PRP administration are still insufficient. Thus far, the clinically acknowledged data on the PRP benefits in skin (patho)physiology include no correlation in between dermal-resident TCs and the beneficial PRP-induced potential for skin regeneration/repair. The growth factors and the substances that PRP locally provides could influence the local dermal TCs into generating the targeted tissue regenerative outcome. Moreover, the presumptive correlation between TCs and PRP could offer new insights into the skin adnexa regeneration processes, or reparatory processes in other organs (Figure 5). However, an interesting idea was promoted with regard to a cellular collaboration between the stem cells and TCs in fulfilling the regeneration process [343].

However, we presume that documenting any possible collaboration between TCs and PRP and the combined administration of PRP and interstitial cells for skin wound treatments, or the regeneration of skin defects, will be a provoking direction for research. Unpublished results have indicated that, in cardiac failure, the heart transplantation of interstitial cell suspensions (enriched in TCs) especially improved cardiac performance. Moreover, there is a current trend for the dermal administering of stimulators of skin’s natural collagen production that gradually contribute to a better-looking appearance of skin. These stimulators are in fact producing local dermal inflammation and probably changes in local dermal TCs that favour inflammation, immune cellular milieu, new blood vessel formation, and finally, collagen production.

## Figures and Tables

**Table 1:** Classification proposed by Dohan Ehrenfest et al. for platelet concentrates based on their cell content and fibrin architecture [249,250].

Category	Name	Description
I	Pure PRP (P-PRP)	Leukocyte-poor preparations with a low-density fibrin network upon activation.
II	Leukocyte and platelet-rich plasma (L-PRP)	Contains leukocytes and forms a low-density fibrin network upon activation.
III	Pure platelet-rich fibrin (P-PRF)	Leukocyte-poor preparations with a high-density fibrin network. Mixed with an activator and requiring a specific separator gel.
IV	Leukocyte and PRF (L-PRF)	Contains leukocytes and has a high-density fibrin network. Blood is centrifuged immediately after collection without the use of anticoagulants, thrombin, or CaCl_2_.

**Table 2:** Studies on acne scar treatments with PRP.

Author(s)	Study Type	Number of Patients	Interventions	Duration and Frequency	Key Findings
Asif et al. [319]	Placebo-controlled, split-face study	50	Micro-needling + PRP vs. Micro-needling alone	Three monthly treatments	62.2% improvement with PRP combination vs. 45.84% in the control group.
Nofal et al. [320]	Randomised, single-blinded, controlled trial	45	PRP injections vs. TCA CROSS technique vs. Micro-needling + PRP	Treatment every 2 weeks for 6 weeks	Significant improvement in all groups compared to baseline, but no difference between the groups at 14 weeks.
Ibrahim et al. [321]	Randomised, comparative trial	90	Micro-needling vs. PRP injections vs. Micro-needling alternating with PRP	Up to six sessions, every 2–4 weeks	Greatest improvement was in the micro-needling + PRP group, followed by PRP alone. Highest patient satisfaction in the combination group.
El-Domyati et al. [245]	Randomised, single-blinded, split-face trial	24	Micro-needling + PRP vs. micro-needling + TCA 15% vs. micro-needling alone	Treatments every 2 weeks, total of six sessions	At 3 months, combination groups showed higher mean scar improvement than micro-needling alone. No significant difference between combination treatments.
Ibrahim et al. [322]	Prospective, single-blinded, split-face clinical trial	35	Micro-needling + PRP vs. micro-needling	Treatments every 3 weeks, total of four treatments	No significant difference between the two groups.
Chawla et al. [317]	Prospective, comparative, split-face trial	27	Micro-needling + PRP vs. micro-needling + topical vitamin C 15%	Treatment every 4 weeks, total of four treatments	At 4 months, the PRP and micro-needling group’s satisfaction rate was higher than that of the micro-needling in association with topical vitamin C group’s. The percentage of patients included in the poor response category was lower in the PRP combination group (22.2%) than in the vitamin C combination group (37%).
Faghihi et al. [323]	Randomised, single-blinded, split-face trial	16	CO_2_ laser + PRP vs. CO_2_ laser	Two monthly treatments	Trend toward improved response with PRP. More erythema and oedema in the PRP group.
Lee et al. [324]	Randomised, split-face trial	14	CO_2_ laser + PRP vs. CO_2_ laser	Two monthly treatments	Faster improvement of laser-induced erythema in PRP group. Shorter mean duration of erythema, oedema, and crusting with PRP.
Gawdat et al. [316]	Randomised, split-face, single-blinded, placebo-controlled study	30	CO_2_ laser + PRP (topical/intradermal) vs. CO_2_ laser	Three monthly sessions	Significant improvement in skin smoothness with PRP. Shorter duration of adverse effects with PRP.
Kar and Raj [325]	Randomised, split-face trial	30	CO_2_ laser + PRP vs. CO_2_ laser	Three monthly sessions	No significant difference in scar scores between PRP and control. Less redness, pain, and swelling with PRP.
Min et al. [326]	Prospective, randomised, single-blinded, split-face trial	25	CO_2_ laser + PRP vs. CO_2_ laser	Two monthly sessions	Greater improvements and patient satisfaction with PRP. Significantly lower side effects with PRP.
Zhu et al. [327]	Clinical study	22	Erbium laser + topical PRP	Three treatments 1–2 months apart	Moderate clinical improvement post-treatment: 68% of patients rated it as excellent or markedly improved. 91% were satisfied or very satisfied.
